# Stressful life events among individuals with a history of eating disorders: a case-control comparison

**DOI:** 10.1186/s12888-021-03499-2

**Published:** 2021-10-13

**Authors:** Selma Ø. Lie, Cynthia M. Bulik, Ole A. Andreassen, Øyvind Rø, Lasse Bang

**Affiliations:** 1 Regional Department for Eating Disorders, Division of Mental Health and Addiction, Oslo University Hospital, Oslo, Norway; 2 Division of Mental Health and Addiction, Institute of Clinical Medicine, University of Oslo, Oslo, Norway; 3 Department of Medical Epidemiology and Biostatistics, Karolinska Institutet, Stockholm, Sweden; 4 Department of Psychiatry, University of North Carolina at Chapel Hill, Chapel Hill, NC USA; 5 Department of Nutrition, University of North Carolina at Chapel Hill, Chapel Hill, NC USA; 6 NORMENT, Institute of Clinical Medicine, University of Oslo, Oslo, Norway; 7 Division of Mental Health and Addiction, Oslo University Hospital, Oslo, Norway; 8 Norwegian Institute of Public Health, Oslo, Norway

**Keywords:** Eating disorders, Bulimia nervosa, Anorexia nervosa, Binge-eating disorder, Trauma, Stressful life events, Risk factor, Adverse events, Sexual abuse, Emotional abuse

## Abstract

**Background:**

Experiencing stressful life events (SLEs) can negatively impact mental health and increase risk for psychiatric disorders including eating disorders (EDs). Previous research has shown that childhood sexual abuse is associated with some EDs, but less is known about the association between other non-sexual SLEs and EDs.

**Method:**

A case-control study of individuals with (*n* = 495, age mean ± SD = 29.1 ± 9.8 years) and without (*n* = 395, age = 30.2 ± 11.7) self-reported lifetime history of EDs was conducted to compare history of self-reported SLEs. Participants reported history of sexual (e.g., rape, other sexual assault) and non-sexual (e.g., emotional abuse, assault, bereavement) life events using an adaptation of the Stressful Life Events Screening Questionnaire. Individuals with EDs were divided into ED subtypes along the restricting – binge eating/purging spectrum to examine subtype differences. Logistic regressions were conducted for each SLE and ED subtype to obtain odds ratios (ORs). We report *p*-values corrected for multiple comparisons.

**Results:**

Exposure to any SLE was significantly more common in individuals with EDs than in controls (OR = 2.47, *p* < .001). Specifically, rape, other sexual assault, and emotional abuse were significantly more common among individuals with a history of binge-eating/purging ED subtypes (ORs = 2.15–3.58, *p*’s < .01) compared with controls. Furthermore, history of life-threatening disease and loss of a close relative/partner/friend were associated with some ED subtypes. The association between SLEs and EDs was stronger for individuals who had experienced multiple SLEs.

**Conclusion:**

By investigating a range of different SLEs, we showed that both sexual and non-sexual SLEs were more common in individuals with a history of EDs (binge-eating/purging subtypes) than controls. Results highlight the importance of assessing a variety of past SLEs in risk assessment for different EDs.

## Introduction

Anorexia nervosa (AN), bulimia nervosa (BN), and binge-eating disorder (BED) are eating disorders (EDs) characterized by dysregulated food intake. Both genetic and environmental risk factors have been implicated in the development and maintenance of EDs [1, 2]. Negative or stressful life events (SLEs) are among many environmental risk factors that have garnered interest in the ED field. In the context of this paper, we consider all negative and potential stressful life events together, including various forms of abuse, assault, bereavement, car accidents, being threatened, been diagnosed with a serious disease, etc. Many of these events are commonly referred to as ‘traumas’, but considering the diversity of events included we collectively refer to these as SLEs. Childhood maltreatment, particularly sexual abuse, is among those most studied and has been implicated as a risk factor for the development of EDs [3–5], and a predictor of treatment dropout [6, 7]. A meta-analysis by Molendijk and colleagues [4] found that childhood maltreatment was two to four times more common among individuals with EDs than healthy controls, and was associated with more severe ED symptoms, earlier onset age, and more frequent binge-eating and purging behaviours.

To date, associations between SLEs and EDs are more consistent for binge-eating/purging subtypes (i.e., BN and BED) than restricting (i.e., AN) subtypes [3, 4, 8–12]. A recent review of published risk factor meta-analyses investigating a range of risk factors found evidence for childhood sexual abuse as a risk factor for BN, while the evidence was weaker for the other ED subtypes [13]. A stronger association between adverse events and binge-eating/purging behaviors has also been reported in individuals with AN, where both childhood and adult sexual abuse were more commonly associated with the binge-eating/purging subtype (AN-BP) than the restricting subtype (AN-R), and was associated with more posttraumatic stress disorder (PTSD) symptoms, emotion dysregulation, and treatment dropout [14–16]. Thus, converging evidence suggests that SLEs, both in childhood and later in life, may be selectively associated with binge-eating/purging EDs. The evidence is more mixed for AN, and few studies directly address differences between AN-R and AN-BP.

Although the majority of research on SLEs in EDs has focused on childhood sexual abuse [5, 17–19], other types of childhood SLEs have also been associated with EDs, including physical and emotional abuse [4, 20, 21]. Studies investigating lifetime SLEs in different populations have found increased risk of ED symptoms in victims of sexual assault [22–24] and in female war veterans who have experienced traumatic events and/or assaults [25]. A range of relatively common childhood events such as parental illness, parental divorce, changing schools, and bullying, have also been asociated with EDs [26–28]. Despite a rich literature highlighting many different types of traumatic or stressful events as significant predictors of risk for mental problems [29], we know little of how common various SLE exposures are among individuals with EDs.

As such, exisiting research supports an association between different SLEs and some EDs, with the most conclusive evidence available for highly traumatic experiences such as sexual abuse. Less is known about other stressful life experiences, and studies investigating other types of SLEs throughout life and their potential associations with different EDs are scarce. Also, few studies have directly investigated how different SLEs are associated with specific EDs (including the two AN subtypes). This has been identified as an important topic for research in recent literature [13, 30], and could have implications for our understanding of the etiology and treatment strategies for EDs.

The current study explored history of SLEs in individuals with a lifetime history of EDs and controls. Specifically, we investigated a) whether exposure to a variety of SLEs differed between individuals with a history of EDs and controls, and b) if the association was stronger for individuals who had experienced multiple types of events. Moreover, we explored differences in SLE exposure across different ED subtypes and report results separately for each subtype compared with controls. The main hypothesis was that SLEs would be more prevalent in individuals with EDs than in controls. We also hypothesised that the association between SLEs and EDs would be stronger for binge-eating/purging subtypes than restrictive subtypes, and that it is more common for individuals with EDs to experience multiple types of SLEs.

## Methods

### Design

The present study used data from the Eating Disorders: Genes & Environment (EDGE) project; a cross-sectional study of Norwegian adults investigating a variety of risk factors for EDs. The study was approved by the Norwegian Regional Committee for Medical and Health Research Ethics (#2017/0606), and all procedures were performed in accordance with ethical guidelines and regulations. All participants provided written informed consent.

### Participants and procedures

Norwegian residents over the age of 16 were invited to participate in the study by completing an online test battery including questions regarding ED history and experiences of SLEs. Individuals with and without lifetime EDs were invited to participate, and recruitment was targeted at user organisations for EDs and specialised ED treatment units in Norway to reach individuals with a lifetime ED. Recruitment through online/social media platforms (e.g., websites, Facebook), and flyers and posters at Norwegian universities targeted both case and control participants. Data were collected between June 2019 and January 2020.

The ED100K (see below) was used to classify cases and controls according to the presence or absence of lifetime DSM-5 EDs [31]. A total of 890 participants were classfied as either cases (*n* = 495) or controls (*n* = 395) according to DSM-5 criteria. Individuals with BN and/or BED (BN/BED *n* = 180) were combined in one group due to the considerable overlap between the two subtypes in lifetime diagnoses. In addition, previous research has found associations between SLEs and the shared symptomatology of these subtypes (i.e., binge-eating), suggesting that there may be underlying processes that are distinct for binge-eating/purging type disorders when compared to EDs characterised by restricting behaviours [4, 32]. AN was divided into the two subtypes (AN-R *n* = 65 and AN-BP *n* = 114) to further explore this hypothesis. A mixed ED group (AN/BN/BED *n* = 133) included all individuals who at some point in their life had met criteria for both AN and BN and/or BED, further emphasising the high crossover between the ED diagnoses over time. We were unable to determine subtype for three individuals in the ED group who were excluded from the subtype analyses. We also calculated the ED onset age, defined as the earliest age of clinically significant symptoms (e.g., low weight, frequent binge eating, compensatory behaviours such as purging, etc.).

### Measures

*ED100K*. The self-report measure ED100K (version 2) was used to assess lifetime history of AN, BN, and BED according to DSM-5 criteria [33]. The ED100K has shown good predictive validity for the different ED types when validated against the Structural Clinical Interview (SCID) for DSM-5 [33], The measure was adapted to fit the study design and was translated into Norwegian (then back-translated to ensure correspondence). The ED100K contains questions about frequency, duration, and severity of core ED features (e.g., weight history, binge eating, compensatory behaviors) and the age of onset for these features. This enabled us to distinguish between ED cases and controls as well as assigning the individuals with EDs to different subtypes.

*Stressful life events screening questionnaire - adapted (SLESQ).* The SLESQ is a validated instrument to assess history of different types of stressful events in clinical and non-clinical populations [34]. The current study used an adapted version of the Norwegian translation developed by Thoresen and Øverlien [35]. Twelve different items (SLEs) were included in the current study; disease (serious/life threatening), accident (serious/life threatening), assault (e.g., physical attack or robbery), bereavement (loss of a close relative, partner, or friend), rape, other sexual assault (unwanted sexual contact/touching), childhood physical abuse (before 18 years of age), adulthood physical abuse (after 18 years of age), emotional abuse, threatened (with a weapon or by threat of force), witnessed (witnessed a situation where another person was hurt, died, or was abused), and other (any other situations of serious threat to life, health, or safety; this item was not specified further). The complete list of questions is included in Table 1. For each of these items, the participants indicated whether they had experienced the event (“yes” or “no”), and at what age (first occurrence). For some items (rape, other sexual assault, adulthood physical abuse, childhood physical abuse, emotional abuse, threatened), we also asked how many times the event in question had occured. In addition to the individual items, all participants who responded “yes” to one or more events were coded as “yes” on an overall “Any SLE” variable.

**Table 1:** Items included in the adapted version of the Stressful Life Events Screening Questionnaire (SLESQ), English translation

Item number	Description
**1: Life-threatening disease**	Have you ever had a life-threatening illness/disease?
**2: Accident**	Were you ever in a life-threatening accident?
**3: Physical assault**	Was physical force or a weapon ever used against you in a robbery or assault?
**4: Bereavement**	Has an immediate family member, romantic partner or very close friend died as a result of accident, homicide, or suicide?
**5: Rape**	Has anyone (parent, other family member, romantic partner, stranger, or someone else) ever forced or threatened you into having intercourse, oral, or anal sex against your will, or when you were in some way helpless?
**6: Other sexual assault**	Other than experiences you have already described, has anyone ever touched your genitals or made you touch theirs against your wishes, or when you were in some way helpless?
**7: Childhood physical abuse**	When you were a child, did a parent, caregiver or other person ever kick you repeatedly, beat or otherwise attack or harm you?
**8: Adulthood physical abuse**	As an adult (> 18 years), have you ever been kicked, beaten, slapped around or otherwise physically harmed by a romantic partner, date, sibling, family member, stranger, or someone else?
**9: Emotional abuse**	Has a parent or a romantic partner systematically ridiculed you, humiliated you, or called you worthless?
**10: Threatened**	Other than the experiences already covered, has anyone ever threatened you with a weapon, like a knife or gun?
**11: Witnessed a traumatic event**	Have you ever witnessed another person being killed, seriously injured, or sexually or physically assaulted?
**12: Other**	Other than the events you have already described, have you ever been in any other situations that was extremely frightening or horrifying, or where you felt very helpless?

*Eating disorder examination-questionnaire (EDE-Q).* The EDE-Q is a self-report measure assessing ED psychopathology in the past 28 days [36], and a Norwegian translation has been previously validated [37]. For the present study, only the global EDE-Q sum scores were used to compare current ED symptoms and behaviours in cases and controls. The measure demonstrated good internal consistency: *α* = .96 for controls and *α* = .95 for the ED group.

### Analysis

For the main analysis, logistic regressions were performed for SLEs comparing each of the ED diagnostic groups with the control group. Odds ratios (ORs) and 95% confidence intervals (CIs) are reported as a measure of effect for each comparison. In all regression models, ED outcome (subtype) was the dependent variable, and the specific SLE (e.g., rape) was entered as a dichotomous predictor (independent variable). We added gender, age, and education as covariates in all models as these variables have been associated with EDs [1], and to reduce recall bias. Unadjusted regression models were conducted without covariates, but these are not reported as the overall pattern of results was similar to the adjusted model.

To investigate whether individuals with EDs had experienced a higher number of SLEs than controls, three levels of SLE exposure were defined: none, one or two types, three or more types. As the measure of SLE number was heavily skewed (majority, > 75%, of participants had experienced between zero and four SLE types), this categorisation was preferred over the continuous measure to specifically compare low (0), medium (1 or 2), and high (≥3) exposure to SLEs while securing relatively even numbers in all groups. This variable was then used to perform logistic regressions comparing the different levels of SLE exposure as predictors for ED outcome (with gender, age, and education as covariates).

Separate analyses of variance (ANOVAs) were used to compare differences in continous variables between case and control groups.

In all models, alpha levels were adjusted using the Bonferroni-Holm correction for multiple comparisons within each family of tests. To ease interpretation, we report corrected *p*-values and *p* < 0.05 was considered statistically significant. All analyses were conducted using R version 4.0.3 [38].

## Results

### Participant characteristics

Table 2 shows sample characteristics. The sample comprised individuals between the ages 16–78 years (M = 29.5 ± 10.6), and was predominantly female (95%). The lifetime ED group did not differ from controls on age or current body mass index (BMI, kg/m^2^ based on self-reported weight and height). The majority (89%) of the ED group reported having received treatment for an ED, and they had a higher EDE-Q global score than controls (*p* < .001). The control group had significantly higher completed education (*p* = .002); more individuals had completed university education ≤4 years in the control group (28.9%) than the ED group (18.4%). The average age of ED onset was 15 years, and the average age reported for any SLE was between 10 and 14 years for the dfferent ED subtypes. Frequency and age for the different SLEs are listed in Table 3.

**Table 2:** Descriptive statistics for individuals with and without lifetime EDs (overall ED and split by subtype)

Lifetime ED status	Any ED (***n*** = 495)		AN-R (***n*** = 65)		AN-BP (***n*** = 114)		BN/BED (***n*** = 180)		AN/BN/BED (***n*** = 133)		No ED (control) (***n*** = 395)	
	M (SD)	Range	M (SD)	Range	M (SD)	Range	M (SD)	Range	M (SD)	Range	M (SD)	Range
**Age (years)**	29.08 (9.76)	16–69	27.20 (9.19)	16–58	27.49 (9.82)	16–66	30.46 (9.71)	16–65	29.70 (9.93)	16–69	30.16 (11.66)	16–78
**EDE-Q global score**	3.32 (1.54)	0–6	2.62 (1.40)	0–5.37	3.50 (1.69)	0.14–5.9	3.19 (1.36)	0.18–6	3.68 (1.59)	0–6	1.28 (1.26)	0–5.72
**Current BMI**	23.85 (7.29)	12.42–58.59	19.97 (2.68)	14.77–32.33	19.78 (3.55)	12.42–38.62	29.45 (8.29)	18.44–58.59	21.51 (4.46)	12.98–46.48	23.94 (4.41)	16.04–48.67
**ED onset age (years)**	15.09 (4.58)	4–50	15.75 (3.41)	6–26	15.39 (4.92)	8–50	14.76 (4.66)	4–35	15.01 (4.28)	6–49	–	–
**Number of SLEs (0–12)**	2.59 (2.31)	0–12	1.74 (2.22)	0–8	2.60 (2.40)	0–12	2.74 (2.26)	0–11	2.85 (2.28)	0–9	1.56 (1.87)	0–11

*Abbreviations*: *AN* anorexia nervosa (AN-R = restricting subtype, AN-BP = binge-eating/purging subtype), *BED* binge-eating disorder, *BMI* body mass index, *BN* bulimia nervosa, *ED* eating disorder, *EDE-Q* Eating Disorder Examination-Questionnaire, *SLE* = stressful life events

**Table 3:** Descriptives and age of stressful life events for individuals with lifetime eating disorders and controls

SLE type	An-R (***n*** = 65)			AN-BP (***n*** = 114)			BN/BED (***n*** = 180)			AN/BN/BED (***n*** = 133)			No ED (***n*** = 395)		
	***n***	%	Age^b^ (years) M (SD)	***n***	%	Age^b^ (years) M (SD)	***n***	%	Age^b^ (years) M (SD)	***n***	%	Age^b^ (years) M (SD)	***n***	%	Age^b^ (years) M (SD)
**Any SLE**^a^	43	66.2	11.4 (7.5)	91	79.8	10.7 (6.5)	153	85	11.5 (7.1)	115	86.5	10.4 (6.7)	255	64.6	14.7 (10.6)
**Disease**	7	10.8	27.9 (16)	18	15.8	17.4 (9.9)	15	8.3	17.6 (15.4)	23	17.3	21.1 (11.2)	30	7.6	27.1 (19.3)
**Accident**	2	3.1	24 (0)	7	6.1	23.3 (10.6)	13	7.2	13.3 (5.4)	9	6.8	19.6 (7.4)	30	7.6	20.4 (14.2)
**Assault**	3	4.6	7.5 (3.5)	11	9.6	18.7 (9.8)	21	11.7	18.7 (7.1)	19	14.3	18.8 (7.0)	24	6.1	20.4 (7.9)
**Bereavement**	5	7.7	13.4 (8.9)	27	23.7	18.3 (8.5)	40	22.2	16.8 (7)	25	18.8	21.4 (9.3)	51	12.9	19 (9.66)
**Rape**	8	12.3	17.3 (10.7)	33	28.9	12.8 (5.9)	57	31.7	15.1 (5.1)	39	29.3	12.3 (6.2)	56	14.2	16.3 (6)
**Other sexual assault**	17	26.2	14.3 (7.8)	41	36	13.2 (5.6)	59	32.8	13.3 (6.1)	47	35.3	13.2 (5.7)	72	18.2	15.7 (6.8)
**Childhood physical abuse**	9	13.8	8 (5.3)	22	19.3	7.3 (4.5)	43	23.9	8.4 (3.9)	29	21.8	7.2 (5.0)	62	15.7	8.1 (4.3)
**Adulthood physical abuse**	7	10.8	19 (8.1)	14	12.3	22.4 (6.3)	31	17.2	20.4 (6.8)	18	13.5	21.8 (2.3)	24	6.1	21.8 (5.7)
**Emotional abuse**	15	23.1	8.1 (5.4)	43	37.7	10.1 (6.6)	80	44.4	11.9 (6.6)	60	45.1	20.2 (8.2)	79	20	13.3 (8.8)
**Threatened**	2	3.1	18.5 (14.9)	8	7.0	16.8 (5.1)	12	6.7	13.8 (7.7)	12	9.0	14.4 (8.8)	24	6.1	20.3 (8.8)
**Witnessed a traumatic event**	5	7.7	10 (3.5)	17	14.9	13.1 (7.9)	17	9.4	12.9 (7.6)	18	13.5	17.1 (13.3)	31	7.8	15.6 (8.7)
**Other**	33	50.8	13.2 (6.6)	55	48.2	13.3 (7.2)	105	58.3	15.7 (8.6)	80	60.2	13.8 (8.3)	132	33.4	18.9 (12.0)

*Abbreviations*: *AN* anorexia nervosa (AN-R = restricting subtype, AN-BP = binge-eating/purging subtype), *BED* binge-eating disorder, *BN* bulimia nervosa, *ED* eating disorder, *SLE* stressful life event

^a^“Any SLE” measures all individuals reporting at least one of the listed SLE types

^b^Age at which the SLE occurred

### Are SLEs more common in individuals with EDs than controls?

A total of 81% of all individuals with lifetime EDs had experienced one or more SLEs, compared to 65% in the control group. Many of the SLEs assessed were events that are commonly experienced throughout a lifetime (e.g., bereavement) and we therefore expected the majority of both cases and controls to report at least one SLE. Of the total ED sample, 56% had experienced one or more SLEs prior to ED onset. Of all the individuals in the ED group who reported a history of SLEs, 68% reported that at least one of the SLEs had occurred prior to ED onset age. The overall case-control comparison of exposure to any SLE in individuals with and without lifetime EDs yielded an OR of 2.47 (95% CI 1.80–3.40, *p* < .001). Individuals in the AN-BP, BN/BED, and AN/BN/BED groups had significantly higher frequency of any SLE than individuals in the control group (all *p’*s < .05; see Table 4). Individuals with AN-R did not differ significantly from controls in overall SLE history (*p* > .05).

**Table 4:** Associations between SLEs and ED subtypes (controlled for gender, education, and age)

SLE type	An-R (***n*** = 65)		AN-BP (***n*** = 114)		BN/BED (***n*** = 180)		AN/BN/BED (***n*** = 133)	
	AN-R vs control OR (95% CI)	***p***-values corrected*	AN-BP vs control OR (95% CI)	***p***-values corrected*	BN/BED vs control OR (95% CI)	***p***-values corrected*	AN and BN/BED vs control OR (95% CI)	***p***-values corrected*
**Any SLE**^a^	1.22 (0.70–2.18)	1.000	**2.29 (1.39–3.91)**	**.017**	**3.01 (1.91–4.88)**	**< .001**	**3.58 (2.11–6.37)**	**< .001**
**Disease**	1.90 (0.71–4.60)	1.000	**2.71 (1.37–5.27)**	**.029**	1.09 (0.54–2.11)	1.000	**2.60 (1.40–4.80)**	**.018**
**Accident**	0.48 (0.08–1.71)	1.000	0.95 (0.36–2.18)	.000	1.04 (0.51–2.04)	1.000	0.93 (0.40–2.00)	.964
**Assault**	0.91 (0.21–2.83)	1.000	1.70 (0.75–3.64)	.923	1.96 (1.03–3.69)	.224	2.45 (1.24–4.81)	.066
**Bereavement**	0.62 (0.21–1.50)	1.000	2.09 (1.20–3.59)	.055	**1.97 (1.23–3.14)**	**.038**	1.75 (1.00–3.02)	.268
**Rape**	0.93 (0.39–2.00)	1.000	**2.17 (1.29–3.61)**	**.029**	**2.59 (1.68–4.00)**	**< .001**	**2.15 (1.32–3.47)**	**.017**
**Other sexual assault**	1.67 (0.87–3.10)	1.000	**2.36 (1.46–3.81)**	**.005**	**2.15 (1.42–3.26)**	**.003**	**2.35 (1.48–3.70)**	**.003**
**Childhood physical abuse**	0.99 (0.42–2.08)	1.000	1.37 (0.77–2.39)	1.000	1.76 (1.11–2.76)	.103	1.56 (0.93–2.60)	.439
**Adulthood physical abuse**	1.18 (0.66–2.09)	1.000	1.06 (0.67–1.67)	1.000	1.05 (0.72–1.52)	1.000	0.80 (0.51–1.23)	.964
**Emotional abuse**	1.32 (0.67–2.47)	1.000	**2.54 (1.57–4.09)**	**.002**	**3.27 (2.20–4.89)**	**< .001**	**3.20 (2.04–5.05)**	**< .001**
**Threatened**	0.52 (0.08–1.93)	1.000	1.10 (0.44–2.50)	1.000	1.03 (0.48–2.11)	1.000	1.45 (0.66–3.05)	.964
**Witnessed a traumatic event**	1.10 (0.36–2.78)	1.000	2.06 (1.05–3.92)	.185	1.19 (0.62–2.22)	1.000	1.71 (0.88–3.21)	.439
**Other**	**2.52 (1.43–4.47)**	**.019**	**2.21 (1.40–3.50)**	**.007**	**2.94 (2.01–4.33)**	**< .001**	**3.10 (2.02–4.80)**	**< .001**

*Abbreviations*: *AN* anorexia nervosa (AN-R = restricting subtype, AN-BP = binge-eating/purging subtype), *BED* binge-eating disorder, *BN* bulimia nervosa, *ED* eating disorder, *SLE* stressful life events

^a^“Any SLE” measures all individuals reporting at least one of the listed SLE types

*p-values corrected for multiple comparisons using Bonferroni-Holm, boldface indicates statistical significance (*p* < 0.05)

### What types of SLEs are associated with the different EDs?

The most commonly reported specified SLE in both ED and control groups was emotional abuse (43 and 21%, respectively). The second most common was other sexual assault (35% of ED group, 19% of controls), followed by rape for the ED group (28% of ED, 14% of controls) and child physical abuse for the controls (22% of ED, 16% of controls). This pattern was similar for each of the ED subtypes, except for AN-R where other sexual assault (26%) was more common than emotional abuse (23%). This is shown in Table 3.

Of all the specified SLEs in the study, three events reached significance (all *p* < .05) for the three groups AN-BP, BN/BED, AN/BN/BED compared with controls: rape, other sexual assault, and emotional abuse (Table 4). Disease was significant for the AN-BP (*p* = .029) and the AN/BN/BED (*p* = .018) groups, and bereavement was significant for the BN/BED group only (*p* = .038). In addition, individuals in all ED groups (including AN-R) scored significantly higher than the control group on the SLE category “other”, which was also the most commonly reported of all the SLE items across all groups.

Follow up analysis for the SLEs reaching significance for the three binge/purge ED groups (rape, other sexual assault, and emotional abuse) revealed no significant differences in age of event between groups except for a slightly lower age for rape in the mixed AN/BN/BED group (M = 12.3 ± 6.2) than controls (M = 16.3 ± 6.0; *p* = .028). The frequency of sexual assault was also higher in the BN/BED (M = 2.5 ± 1.19) group than in controls (M = 1.59 ± 0.86; *p* < .001). No other group differences in frequency were significant. In the ED sample as a whole, the proportion of reported SLEs that had occurred prior to ED onset age was 43.1% for rape, 56.6% for emotional abuse, and 49.4% for other sexual assault.

### Is the association between SLEs and EDs stronger for individuals with a history of multiple SLEs?

Individuals with EDs reported experiencing a higher number of different SLEs (M = 2.6, M_dn_ = 2) than individuals in the control group (M = 1.6, M_dn_ = 1; *p* < .001). The overall case-control comparison showed that individuals in the ED group were significantly more likely to have experienced three or more SLEs (OR 2.08, 95% CI 1.49–2.90), than one or two SLE types (OR 1.82, 95% CI 1.30–2.57) compared with controls (see Table 5). Apart from the AN-R subtype, a pattern emerged that was consistent with a cumulative effect of multiple SLE types for all subtypes (AN-BP, BN/BED, AN/BN/BED). Thus, there was support for an association between binge-eating/purging EDs and SLEs that was stronger for individuals with more extensive SLE history.

**Table 5:** Associations between multiple SLEs and ED groups (overall and by subtype) versus controls (controlled for age, gender, and education)

Diagnosis	No SLEs	One or two SLEs	Three or more SLEs	One or two SLEs vs no SLEs		Three or more SLEs vs one or two SLEs	
	***n*** (%)	***n*** (%)	***n*** (%)	OR (95% CI)	***p***-values corrected*	OR (95% CI)	***p***-values corrected*
No ED (control)	140 (35%)	167 (42%)	88 (22%)	–	–	–	–
Any ED^a^	92 (19%)	192 (39%)	211 (43%)	**1.82 (1.30–2.57)**	**.004**	**2.08 (1.49–2.90)**	**< .001**
AN-R	22 (34%)	28 (43%)	15 (23%)	1.17 (0.64–2.17)	1.000	1.14 (0.56–2.27)	1.000
AN-BP	23 (20%)	39 (34%)	52 (46%)	1.52 (0.87–2.73)	.446	**2.58 (1.55–4.34)**	**.002**
BN/BED	27 (15%)	69 (38%)	84 (47%)	**2.13 (1.30–3.57)**	**.017**	**2.24 (1.48–3.42)**	**.001**
AN/BN/BED	18 (14%)	55 (41%)	60 (45%)	**2.71 (1.54–4.99)**	**.005**	**1.94 (1.22–3.10)**	**.021**

*Abbreviations*: *AN* anorexia nervosa (AN-R = restricting subtype, AN-BP = binge-eating/purging subtype), *BED* binge-eating disorder, *BN* bulimia nervosa, *CI* confidence interval (95%), *ED* eating disorder, *OR* odds ratio, *SLE* stressful life event

^a^“Any ED” refers to all individuals with a lifetime ED (any subtype)

*p-values corrected for multiple comparisons using Bonferroni-Holm, boldface indicates statistical significance (*p* < 0.05)

## Discussion

The current study investigated the history of a variety of different lifetime SLEs in individuals with and without lifetime EDs, differentiated by subtype. Individuals with binge-eating/purging subtypes of EDs had experienced SLEs more often than controls, and it was more common for these groups to be exposed to multiple types of events. Rape, other sexual assault, and emotional abuse were significantly more common in the ED group as a whole than in the control group, and this held true for all ED subtypes with the exception of AN-R. This is consistent with previous research reporting a stronger association between a range of SLEs and binge-eating/purging EDs than restrictive AN [4, 12, 26, 39, 40]. The event category “other SLE” was significantly more common in all ED groups, and certain SLEs (bereavement and life-threatening disease) were more common for only some ED subtypes. The average onset age for both ED onset and SLE was during adolescence, and the majority of SLEs in the ED group occurred prior to ED onset. More than half of the ED group had experienced at least one SLE prior to developing significant ED symptoms, which raises the possibility that such events can be a trigger contributing to the onset of EDs. By assessing a number of different SLEs throughout life, we showed that both non-sexual and sexual SLEs are associated with binge-eating/purging EDs and thus add to the growing knowledge of sociocultural factors in EDs.

Our findings support the observation that exposure to SLEs is more prevalent among individuals with binge-eating/purging EDs than controls. These individuals were between two and three times more likely than controls to have experienced any SLE, with the highest individual associations being for sexual and emotional abuse. The association was also stronger for those with a higher number of different SLEs. This finding is consistent with other research showing a cumulative effect of multiple adversities on a range of negative health outcomes [41]. The strength of the associations in the present study is comparable to previous studies finding OR’s in the range of 2–3 for different types of adversities including emotional, physical and sexual child abuse, sexual assault, family disruption, parental psychiatric illness, parental teasing, and bullying [3, 26, 40, 42–45]. We have previously reported associations between school-age bullying and EDs in the same sample as the present study, with a similar pattern of results [28]. Together, these findings indicate that history of SLEs is common among individuals with EDs, underscoring the importance of environmental factors in these patient groups.

Various SLEs were more common in individuals with AN-BP, BN/BED, and AN/BN/BED than controls, whereas this was not the case for the AN-R group. While we note that these analyses could be underpowered due to a relatively small sample size for the AN subtypes (*n* = 65 for AN-R, *n* = 114 for AN-BP), other studies have found similar subtype differences regarding AN-R [14, 16]. Why individuals with restricting ED subtypes would be less likely to have experienced SLEs is unclear. One interpretation is that SLEs and traumas cause behaviors that are characterized by impulsivity, as many studies have found associations between SLEs and other impulsive behaviours and maladaptive coping such as suicide attempts [46] and substance abuse [47, 48] in addition to the more ED specific behaviours binge eating and purging [4, 10, 49]. Behavioural impulsivity has also been shown to mediate the relationship between childhood abuse and binge eating and purging in non-clinical populations [50, 51]. Relatedly, personality traits such as sensation seeking and disinhibition have been associated with both BN/BED and victimisation experiences [52]. In addition, SLEs might differentially affect BN and BED – and different types of SLEs could be related to specific types of binge/purge symptoms. This would be obscured due to the combination of BN and BED in one group in the current study, and would need to be explored in future research.

In line with findings implicating an association between impulsive psychopathologies and SLEs, it has been suggested that AN-R in particular might have a different etiology than other EDs [1, 26, 53]. Different genetic pathways and interactions between genetic profile and traumatic events could thus account for the observed difference between restricting and binge-eating/purging EDs [54–56]. Genetic research on AN has also found both psychiatric and metabolic genetic correlations [57], and it is noteworthy that heritability estimates are higher in restricting than binge-eating/purging EDs, possibly suggesting a more biological etiology [58]. These observations along with the findings in the present study, may indicate that environmental stressors and triggers are more important in the etiology of binge/purge ED subtypes than restricting subtypes, which could have clinical implications for prevention and treatment.

In terms of specific SLEs, our results highlight that a range of SLEs are commonly experienced by individuals with EDs. This is in line with previous findings (e.g., [4, 59]). Sexual traumas (rape and other sexual assault) were significantly associated with binge-eating/purging ED subtypes in the current study. This is consistent with previous literature highlighting sexual abuse in childhood [3–5] and sexual assault and harassment in adulthood [23, 42, 60–63] as risk factors for ED psychopathology and diagnosis. We also found that emotional abuse was significantly associated with binge-eating/purging EDs. This was common for both cases and controls (20–45%), and resulting ORs were particularly high (2.54–3.27). Emotional abuse has previously been studied mainly in childhood and adolescence and although fewer studies are available than for sexual abuse, the research supports a role of childhood emotional abuse in EDs [4, 16, 20, 21]. Additionally, bereavement was significantly associated with BN/BED, which is consistent with a recent review highlighting non-abusive family-related risk factors [64]. Having a serious or life-threatening disease was significantly associated with AN-BP and AN/BN/BED. This could be consistent with previous research implicating immune system disturbances as risk factors for EDs [13, 65, 66], but we cannot rule out the possibility that some participants might have responded “yes” to this item because of their ED—for example in the case of somatically unstable AN or other complications. The final SLE category “other stressful life events” was also significant for all ED groups. This could include a range of different life experiences (for example divorce, financial difficulties, etc.), and indicates that there could be many more SLEs other than «typical» traumatic experiences that could be of interest to investigate in ED populations.

On average, most of the significant events occurred during early to middle adolescence in the present study. Thus, the average age for the significant SLEs associated with EDs was between 10 and 21 years for the different diagnostic groups, indicating that many of these events would have coincided with ED onset age (average 15 years). Indeed, in 68% of the individuals with EDs who reported SLEs, at least one event occured prior to our calculated ED onset age based on presence of symptoms. This is consistent with the notion that these events could be risk factors for the development of EDs [2], and in line with a handful of longitudinal studies finding increased risk of disordered eating in individuals who have experienced childhood maltreatment or other traumas [67–70]. Moreover, in a study by Brewerton et al. [71], history of victimisation and PTSD symptoms were associated with child onset binge eating, as opposed to adult onset. Thus, the timing of ED onset and SLE occurrence is an important factor to include in future studies. As there are still unanswered questions regarding the mechanisms of the associations between EDs and SLEs, further prospective studies are needed to explore this and extend to a wider range of lifetime experiences.

Although childhood physical abuse has previously been linked to EDs [3, 4], both childhood and adulthood physical abuse were among the SLEs not significicantly associated with EDs in the present study. Some of these non-significant associations could be due to low power, as some events were infrequent in both ED and control groups. For example, being threatened was only experienced by 3–9% of individuals in all groups. The low prevalence for some events can be due to cultural and societal factors, as for example childhood maltreatment is less common in Scandinavian countries compared to the US and many other countries [72–74]. As such, we contribute to the current understanding of stressful life events and EDs by highlighting the role of sexual and emotional stressors, while not ruling out the possibility that other types of events may be important.

The current study used a convenience sample, and recruited participants mainly through online channels. Many of the participants were reached through ED-specific social media accounts and user-forums. This might have biased our sample towards more severe ED presentations, with a higher proportion of AN in the ED case group than what we would expect in the general population. In addition, this sampling method is likely to reach predominantly younger participants, and the sample had an average age of ≈ 30 yrs. (Mdn = 27). We therefore acknowledge that the ED case groups in the current study may not be representative of the ED population at large. However, as previous research has also found, there seems to be a subgroup of individuals with EDs who have a history of SLEs that may be related to the development, maintenance, severity, or clinical presentation of their symptoms. The co-occurrence and relationship between ED symptoms and SLE history has implications for treatment strategies for this group, and we refer to other studies providing in-depth discussions and experiences about the clinical interventions and treatment delivery for these individuals [75–77].

Our study has a number of limitations. First, we used self-report measures both for establishing ED status and for documenting SLEs. However, we used a comprehensive and previously validated measue (ED100K) for ED assessment and a validated measure for SLEs. Second, the retrospective nature of our study may introduce recall bias, which we attempted to account for by controlling for current age. This design also precludes us from drawing causal inferences, and further studies are needed to explore potential bi-directional relationships or third-variables that could affect the associations between SLEs and EDs. For example, we did not measure PTSD symptoms in the current study and we cannot determine whether the observed differences were influenced by such symptoms. Third, we did not consider events such as for example military experiences or natural disaster, and there could be other events that are associated with EDs not explored in the current study. Fourth, we did not include a psychiatric control group, and the sample was predominantly female. We also lack information regarding race or ethnicity of our sample, but note that the population of Norway is primarily of Northern European descent. Last, we did not directly compare ED subtypes as these comparisons would likely suffer from low power, and we combined BN and BED into one group which prevented us from investigating potential differences in SLE history for these two subtypes.

## Conclusions

The current study showed that SLEs were more frequently reported by individuals with binge-eating/purging EDs than controls. While previous studies of SLEs have typically focused on sexual abuse, we queried a number of different events and found that both sexual (e.g., rape, other sexual assault) and non-sexual (e.g., emotional abuse, bereavement) events were more common among individuals with a history of EDs. These events may consistute risk factors, but prospective studies are needed to establish this further. Future studies are also needed to explore if timing of events is important, and whether there is a dose-response relationship between SLEs and EDs. In addition, an interesting avenue for further research is the exploration of potential gene – environment interactions, which may be relevant in the study of risk factor assessment for EDs [78]. Our results highlight that one or more SLEs are more commonly reported in individuals with ED subtypes marked by binge eating and purging than in controls, and encourage thorough assessment of SLEs to inform case conceptualization, treatment strategies, and risk assessment for this patient group.

## Data Availability

The datasets used and/or analysed during the current study are available from the corresponding author on reasonable request.

## Abbreviations

AN-BP: Anorexia nervosa binge-eating/purging subtype
AN-R: Anorexia nervosa restricting subtype
BED: Binge-eating disorder
BN: Bulimia nervosa
ED: Eating disorders
OR: Odds ratio
SLE: Stressful life event
